# Does C-reactive protein exhibit high prognostic information value in acute pulmonary embolism? A novel structural pathway for disease progression beyond classical statistical associations

**DOI:** 10.1371/journal.pone.0343108

**Published:** 2026-02-18

**Authors:** Andrzej Tukiendorf, Piotr Feusette

**Affiliations:** 1 Institute of Health Sciences, Opole University, Opole, Poland; 2 Institute of Medical Sciences, Opole University, Opole, Poland; Osaka University Graduate School of Medicine, JAPAN

## Abstract

Acute pulmonary embolism (APE) is a life-threatening condition requiring precise risk stratification. Although numerous prognostic factors have been proposed, redundancy and limited predictive utility often obscure clinical interpretation. To analyze a predefined set of clinical and laboratory variables in patients with APE using both classical statistical models and a novel taxonomic structural analysis, aiming to identify factors associated with early mortality beyond conventional outcome-based associations. We retrospectively analyzed 366 patients diagnosed with APE between 2009 and 2018, of whom 76 died within one year of the acute event. A total of 20 clinical and laboratory variables—including both established prognostic markers and features with no presumed direct impact on mortality—were assessed using Cox and logistic regression models with the concordance index (C-index) and Akaike’s Information Criterion (AIC). A structural analysis based on Marczewski–Steinhaus (M–S) taxonomic distances was applied to all 1,140 unique triads of risk factors to identify clusters of high patient variability. Segmented regression was then used to determine the transition between homogeneous and heterogeneous predictor spaces. Classical regression identified age as the strongest mortality predictor in APE. In contrast, the taxonomic outcome-agnostic approach revealed CRP as the most prominent structural signal, followed by other key inflammatory markers such as D-dimer, high-sensitivity troponin T (hsTnT), and activated partial thromboplastin time (aPTT). Age, along with certain hematological parameters (e.g., hemoglobin) and major electrolytes (Na ⁺ , K ⁺ , Cl⁻), appeared taxonomically insensitive to acute disease-related changes, reflecting more stable background characteristics. Several other variables, including renal biomarkers (urea, creatinine, and GFR), showed no significant role in APE, with their levels varying randomly between patients. Within this framework, CRP exhibits the highest structural variability among the analyzed factors, suggesting prognostic relevance beyond classical outcome-based associations (such as age). The proposed taxonomic approach complements traditional methods by reducing redundancy, enhancing interpretability, and improving the identification of truly relevant prognostic factors.

## Introduction

Acute pulmonary embolism (APE) is a serious and potentially life-threatening condition. Its clinical significance stems from its high mortality rate, the need for accurate risk stratification, diverse clinical presentation, diagnostic complexity, treatment challenges, and implications for long-term management [1]. Identifying key prognostic variables is therefore crucial. These include hemodynamic instability, right ventricular dysfunction, cardiac biomarkers (e.g., troponins, natriuretic peptides), demographic factors (e.g., age, comorbidities), inflammatory markers, imaging findings, laboratory values (e.g., white blood cell count, fibrinogen-to-albumin ratio), and composite clinical scores such as the Pulmonary Embolism Severity Index (PESI) [2–5].

The high dimensionality of clinical datasets necessitates robust variable selection techniques to reduce redundancy and improve predictive performance [6]. Redundant predictors—particularly in cardiovascular datasets—may reflect internal clustering and compromise model interpretability [7]. Studies have shown that predictive accuracy can often be maintained even when the number of predictors is drastically reduced (e.g., from 433 to 38 [8]); however, this does not necessarily imply that the retained variables are the most clinically meaningful. This highlights the need for approaches that assess the structural relevance of variables beyond their predictive contribution [8].

In this study, we introduce a taxonomic, outcome-agnostic framework for evaluating prognostic variables. This approach assesses structural interrelationships between variables, regardless of their direct associations with clinical endpoints. By applying a matrix of Marczewski–Steinhaus distances and segmented regression, we aim to identify variables with high informational impact that may be overlooked by classical statistical models [9,10].

## Materials and methods

### Ethics approval

The study was approved by the Bioethics Committee at the Opole Chamber of Physicians (Resolution No. 268, dated May 17, 2018). The Committee granted permission to use anonymized clinical data of patients diagnosed with acute pulmonary embolism for scientific research purposes, without the requirement to obtain individual informed consent, provided that patient confidentiality is maintained. The study was conducted in accordance with the principles of the Declaration of Helsinki.

### Study population and data sources

This study included all patients diagnosed with acute pulmonary embolism (APE) who were treated at the Department of Cardiology, University Clinical Hospital in Opole, between December 2009 and December 2018. The dataset comprised medical records of 366 consecutive individuals with confirmed APE (150 [41%] male, 216 [59%] female; mean age, 65.0 ± 16.6 years; median, 68; range, 19–94). A broad range of clinical data was collected, including demographic information, comorbidities, imaging results (echocardiography and ultrasound), and laboratory findings—yielding nearly one hundred parameters used for clinical characterization.

APE was diagnosed based on the following criteria:

- Chest computed tomography pulmonary angiography (CTPA) showing a filling defect in the pulmonary arteries, accompanied by clinical symptoms such as dyspnea and chest pain (325 patients, 89%);
- Characteristic clinical presentation, elevated D-dimer levels, and visualization of a thrombus in the right heart chambers (20 patients, 6%);
- Positive Doppler ultrasound of the lower limb veins in conjunction with symptoms and elevated D-dimer levels (15 patients, 4%);
- Abnormal pulmonary scintigraphy findings, supported by clinical symptoms and elevated D-dimer levels (4 patients, 1%).

Patients without objective imaging confirmation or consistent clinical and laboratory features were excluded from the study.

Mortality data were retrieved from the regional database of the National Health Fund (Opole branch), with 76 deaths observed within one year after the APE event.

The remaining clinical data were retrieved from the medical records of the University Clinical Hospital in Opole, Poland. All data were accessed for research purposes between 03/07/2023 and 29/12/2023.

We evaluated 20 potential prognostic variables: age, body mass index (BMI), hemoglobin (Hb), white blood cell count (WBC), red blood cell count (RBC), platelet count (PLT), urea (UREA), creatinine (CREA), glomerular filtration rate (GFR), glucose (GLU), sodium (Na⁺), potassium (K⁺), chloride (Cl⁻), CK-MB mass, high-sensitivity troponin T (hsTnT), D-dimer, adjusted D-dimer (aD-dimer), international normalized ratio (INR), activated partial thromboplastin time (aPTT), and C-reactive protein (CRP). In addition to clinically established prognostic markers, variables with no presumed direct impact on APE-related mortality (e.g., sodium, chloride) were deliberately included to enhance the overall variability of the dataset.

Table 1 summarizes the descriptive statistics for all evaluated variables, including measures of central tendency (mean and median), dispersion (standard deviation [SD] and interquartile range [IQR]), coefficient of variation (CV), and the results of the Kolmogorov–Smirnov test for normality (where a p-value > 0.05 indicates no significant deviation from a normal distribution).

**Table 1:** Descriptive statistics for evaluated risk factors, including mean, median, standard deviation (SD), interquartile range (IQR), coefficient of variation (CV), and p-value from the Kolmogorov–Smirnov normality test.

Risk factor	Mean	Median	SD	IQR	Min	Max	CV	P-value
Age	65.0	68.0	16.6	22.0	19.0	94.0	0.26	0.0052
BMI	29.5	29.4	5.87	7.24	15.4	52.0	0.20	0.1075
Hb	13.4	13.5	1.82	2.40	7.80	17.9	0.14	0.8427
WBC	11.1	10.3	4.19	5.11	3.10	35.6	0.38	0.0002
RBC	4.57	4.59	0.65	0.87	2.56	6.50	0.14	0.9991
PLT	215	205	82.6	93.8	8.40	681	0.38	0.0097
UREA	41.5	36.3	21.3	22.1	7.50	147	0.51	<0.0001
CREA	1.02	0.95	0.39	0.42	0.34	2.81	0.38	<0.0001
GFR	84.1	78.0	40.6	55.8	9.00	259	0.48	0.0148
GLU	143	120	69.3	54.8	65.0	595	0.48	<0.0001
Na+	138	139	3.67	4.00	125	152	0.03	0.0004
K+	4.30	4.26	0.53	0.57	2.66	6.34	0.12	0.0145
Cl-	101	102	4.28	5.00	84.0	114	0.04	0.0019
CK-MB mass	4.70	3.36	5.79	3.55	0.31	61.4	1.23	<0.0001
hsTnT	123	45.7	232	107	3.00	2011	1.88	<0.0001
D-Dimer	11193	6475	11407	9914	97.0	54982	1.02	<0.0001
aD-Dimer	26.1	11.8	67.1	15.9	0.16	755	2.57	<0.0001
INR	1.20	1.07	0.73	0.17	0.87	12.9	0.61	<0.0001
aPTT	42.9	29.6	42.1	11.3	15.8	222	0.98	<0.0001
CRP	40.7	22.5	50.0	42.1	0.67	296	1.23	<0.0001

Table 1 reveals substantial variability across the examined variables, with adjusted D-dimer (CV = 2.57), hsTnT (CV = 1.88), and CRP (CV = 1.23) exhibiting the highest relative dispersion. In contrast, sodium (Na⁺) showed the lowest variability (CV = 0.03). According to the Kolmogorov–Smirnov test, most variables significantly deviated from a normal distribution (p < 0.05), with only a few—such as hemoglobin and red blood cell count—demonstrating approximately normal characteristics.

**Fig 1:**
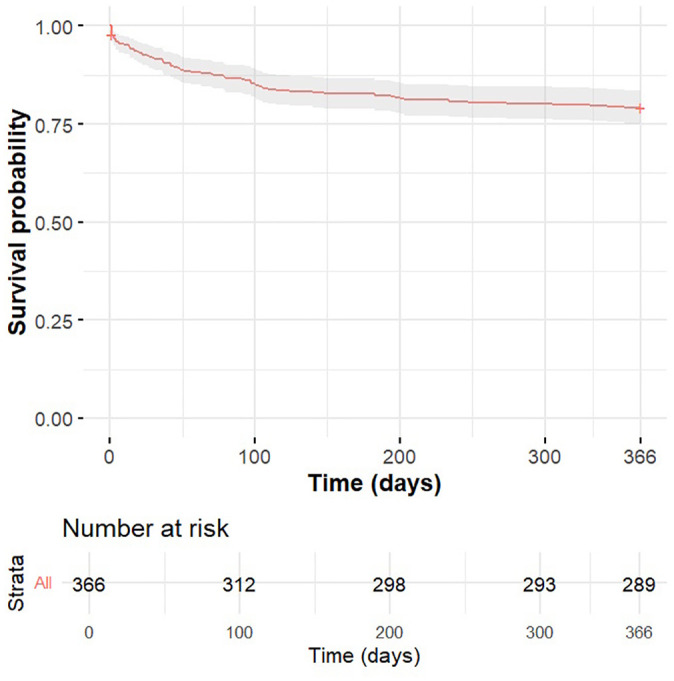
Kaplan–Meier survival curve for the study cohort over the 1-year follow-up period. The shaded area indicates the 95% confidence interval. The risk table below the graph shows the number of patients remaining under observation at selected time points.

Overall survival probability during the 1-year follow-up period was estimated using the Kaplan–Meier method (Fig 1).

The survival curve presented in Fig 1 demonstrates a gradual decline in survival probability, with the most pronounced decrease occurring within the first few days following admission. Thereafter, the curve levels off, indicating a slower rate of mortality in the subsequent months. Notably, the median survival time was not reached during the one-year follow-up period.

Model discrimination and parsimony were assessed using the concordance index (C-index), Akaike’s Information Criterion (AIC), and p-values. In Cox regression, the C-index quantifies the model’s ability to correctly rank survival times, ranging from 0.5 (no better than chance) to 1.0 (perfect discrimination). The AIC facilitates model comparison by balancing goodness-of-fit with model complexity and is calculated as AIC = 2k – 2ln(L), where k is the number of model parameters and L is the maximized likelihood function.

Lower AIC values indicate better-fitting models with fewer parameters. In both Cox and logistic regression analyses, the statistical significance of a risk factor’s association with the clinical outcome was assessed using the p-value, with p < 0.05 considered statistically significant and higher values indicating insufficient evidence of association.

In our proposed structural analysis of predictors, based on Newton’s binomial theorem, we considered all ((20 3) =) 1,140 unique triads (combinations without repetition) of 20 clinical variables. For each triad, square matrices of pairwise taxonomic distances between patients (symmetric dissimilarities) were computed using the Marczewski–Steinhaus (M–S) metric [10]. In this approach, the symmetric taxonomic distance (D) between two subjects (A and B) is defined as:


D=|A−B|max(A, B)

where the numerator represents the absolute difference between A and B, and the denominator is their maximum value [10].

This metric has been successfully applied in several prior studies (e.g., [11]). Notably, one such study demonstrated that the seemingly simple M–S metric produced classification results in striking agreement with those generated by the more complex Expectation–Maximization (E–M) algorithm [12]—which, incidentally, was reported in 2014 to be the second most cited statistical paper globally, following Sir D. R. Cox’s seminal 1972 article on regression models [13,14].

The foundational concept of the M–S metric was later highlighted by Stanisław Marcin Ulam—Steinhaus’s student and collaborator, participant in the renowned Scottish Café gatherings, and a key contributor to the Manhattan Project—who noted its usefulness in practical and biological applications [15].

The Marczewski–Steinhaus (M–S) distance was selected because it measures relative rather than absolute differences between objects. Unlike Euclidean distance, which aggregates squared deviations across all dimensions, M–S focuses on the proportion of difference to the maximum observed value within each pairwise comparison. This makes it inherently scale-invariant after standardization and more appropriate when the interest lies in structural dissimilarity patterns rather than magnitude alone. Moreover, M–S satisfies the triangle inequality while preserving metric properties even in cases where variables have sparse or zero-inflated distributions—conditions under which Euclidean distance can artificially inflate separation. The original formulation by Marczewski and Steinhaus also emphasized its adaptability for set-theoretic interpretations, allowing the same measure to be meaningfully applied to both numerical and categorical data, which broadens its applicability in heterogeneous clinical datasets.

Then, we generated a scree plot of triads ranked by average distance and applied segmented regression to detect a breakpoint distinguishing low- from high-variability segments (the division into two segments was chosen to avoid complexity and allow clearer interpretation of the statistical findings).

In each defined segment, the frequency of occurrence for each clinical variable was calculated across all ((19 2) =) 171 possible triads in which it was present, complemented by the remaining (1140 − 171 =) 969 triads in which it was absent.

The frequency of each variable’s appearance across segments was calculated, and chi-square tests identified significant overrepresentation.

All statistical computations were performed using R software [16], with the aid of the ‘survminer’, ‘cluster’, and ‘segmented’ packages for survival analysis, taxonomic clustering, and regression segmentation, respectively [17–19].

### Methodological note

In summary, the taxonomic approach applied in this study differs fundamentally from conventional regression-based methods. While classical models (e.g., Cox or logistic regression) rely on outcome-dependent associations and require explicit model assumptions, our method is unsupervised and outcome-agnostic. By calculating Marczewski–Steinhaus (M–S) distances within triads of clinical variables, it quantifies structural divergence between patients in a purely geometric sense. This allows for the detection of latent heterogeneity that may be masked by additive or linear assumptions. Unlike subject-based analyses, this approach operates on combinations of variables, evaluating their behavior across all possible triads. As a result, it enables the identification of variables that make the largest contribution to structural variability within the dataset.

## Results

The statistical analyses yielded results from both classical regression models and the taxonomic approach, enabling a comparison between outcome-dependent and outcome-agnostic perspectives. Table 2 presents the findings from classical regression analyses, including coefficients of variation, p-values from Cox and logistic regressions, concordance indices (C-index), and Akaike’s Information Criterion (AIC) values from both univariate and multivariate models.

**Table 2:** Coefficients of variation, p-values from Cox and logistic regression, concordance index (C-index), and Akaike’s Information Criterion (AIC) obtained from univariate and multivariate analyses.

Rrisk factor	CV	Cox regression p-value (univariate)	C-index	Cox p-value (multivariate)	Logistic regression p-value (univariate)	AIC	Logistic regression p-value (multivariate)
Age	0.26	<0.0001	66.6%	0.0089	<0.0001	350.71	0.0075
BMI	0.20	0.1140	56.5%	0.1474	0.1140	375.32	0.2494
Hb	0.13	0.0144	57.4%	0.0458	0.0171	372.16	0.0997
WBC	0.38	0.0097	57.5%	0.9508	0.0201	372.65	0.8535
RBC	0.14	0.2030	53.5%	0.1030	0.2000	376.28	0.2048
PLT	0.38	0.6080	49.5%	0.5858	0.6320	377.70	0.4617
UREA	0.51	<0.0001	65.2%	0.1029	<0.0001	358.09	0.1213
CREA	0.38	0.0017	56.0%	0.8144	0.0074	370.96	0.6613
GFR	0.48	<0.0001	66.2%	0.8718	<0.0001	356.34	0.7248
GLU	0.48	0.0004	64.4%	0.0675	0.0040	369.76	0.2498
Na+	0.03	0.0361	59.1%	0.2576	0.0666	374.58	0.4230
K+	0.12	0.0304	55.9%	0.3076	0.0376	373.64	0.1794
Cl-	0.04	0.0026	59.9%	0.1536	0.0083	370.95	0.1334
CK-MB mass	1.23	0.0001	62.5%	0.1784	0.0030	365.85	0.1499
hsTnT	1.88	0.0007	65.5%	0.5163	0.0021	367.27	0.4750
D-dimer	1.02	<0.0001	63.1%	0.0604	0.0001	361.97	0.0510
aD-dimer	2.57	0.3390	58.5%	0.2065	0.3440	377.10	0.3636
INR	0.61	0.0001	61.7%	0.0009	0.0357	370.63	0.1242
aPTT	0.98	0.2580	53.6%	0.9143	0.2570	376.72	0.8114
CRP	1.23	0.0019	63.1%	0.7829	0.0056	370.54	0.5565

Table 2 shows that Cox regression identified 15 variables as statistically significant (p < 0.05), while logistic regression identified 14. Among them, age emerged as the most predictive factor (C-index = 66.6%, AIC = 350.71), whereas platelet count demonstrated the weakest discriminatory performance (C-index = 49.5%, AIC = 377.70). In multivariate Cox regression, age, hemoglobin, and INR remained significant; in multivariate logistic regression, only age retained statistical significance.

**Fig 2:**
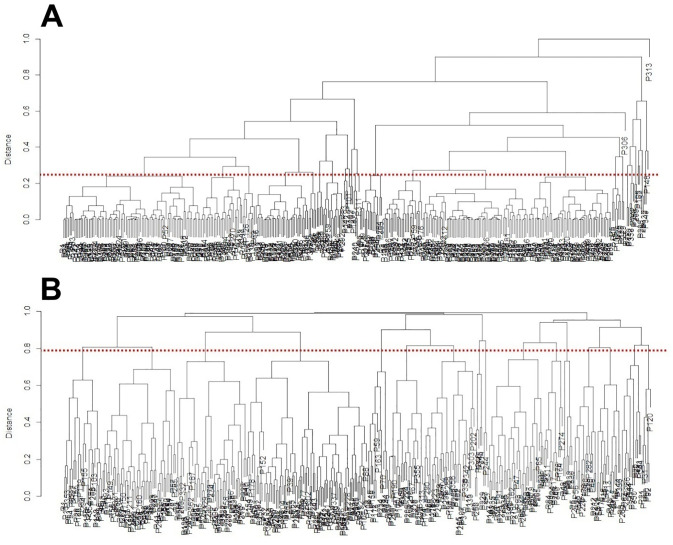
Dendrograms representing the lowest (Panel A) and highest (Panel B) average taxonomic distances among the 1,140 triads of clinical variables. Panel A. The most structurally cohesive triad: Na⁺/Cl⁻/INR (mean taxonomic distance = 0.259). Panel B. The most structurally divergent triad: hsTnT/adjusted D-dimer/CRP (mean taxonomic distance = 0.709).

The taxonomic analysis identified the triad Na ⁺ /Cl ⁻ /INR as having the lowest average structural distance (0.259), while the triad hsTnT/adjusted D-dimer/CRP showed the highest average distance (0.709), as illustrated in Fig 2, Panels A and B, respectively.

In the dendrograms shown in Fig 2, individual patients (labeled as “P” followed by identification numbers) are hierarchically clustered based on nearest-neighbor relationships derived from Marczewski–Steinhaus distances. Each leaf represents a single patient, and patients that are more similar in the three variables forming a given triad are joined together at lower levels of the tree, while more dissimilar patients merge at higher levels. Thus, the vertical height at which branches join reflects the magnitude of taxonomic distance between patients: short branches indicate structural similarity, whereas long branches indicate pronounced divergence in clinical profiles. The red dashed horizontal line marks the mean taxonomic distance for a given triad and serves as a reference level separating relatively homogeneous patient groupings from more dispersed ones. Triads whose dendrograms extend far above this line therefore represent variable combinations that generate high inter-patient variability, suggesting that these variables are strongly involved in differentiating patients in a disease-relevant manner. Conversely, triads with compact dendrograms below this line indicate more uniform, stabilizing variables that contribute little to structural heterogeneity.

This visualization allows the reader to directly see how different combinations of clinical variables either compress patients into similar profiles or spread them apart into highly heterogeneous patterns—an effect that is not accessible from regression coefficients alone.

**Fig 3:**
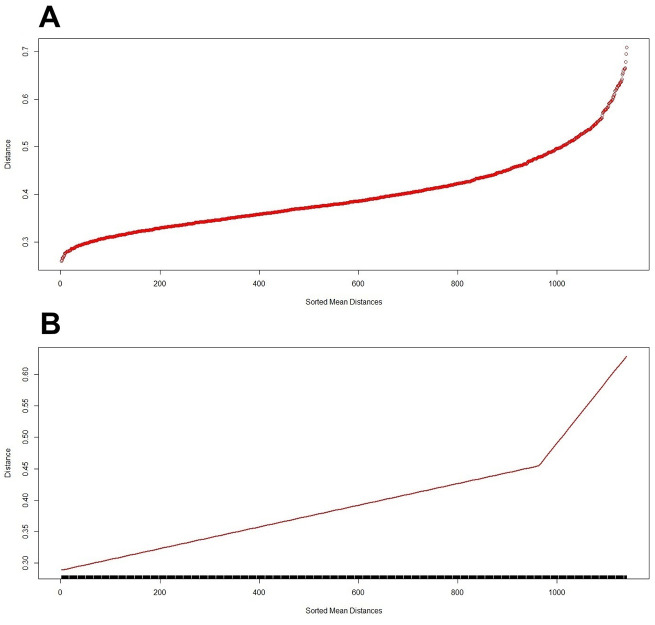
Scree plot of average taxonomic distances derived from 1,140 triads of 20 clinical risk factors (Panel A), and the corresponding segmented regression model fitted to identify the change-point in structural variability (Panel B). Panel A. Distribution of average taxonomic distances across all triads. Panel B. Segmented regression identifying structural change-point.

The scree plot of average taxonomic distances across all 1,140 triads formed from 20 clinical risk factors is shown in Fig 3, Panel A. Panel B displays the corresponding segmented regression model used to identify a structural change-point in the distribution.

As detailed in Table 3 and illustrated in Fig 2, Panel B, a distinct change-point was identified between triads 963 and 964, corresponding to an average taxonomic distance of 0.478.

**Table 3:** Segmented regression results for average taxonomic distances across 1,140 triads of clinical risk factors.

Regression parameter	Mean	Confidence interval 95%	p-value
slope1	0.000172	(0.000170,0.000174)	<0.0001
slope2	0.000988	(0.000962,0.001013)	<0.0001
change-point	963.2		

As shown in Table 3, triads ranked 1 through 963 define the first segment, characterized by modest increases in average taxonomic distance (‘slope1’), whereas the remaining 177 triads (ranks 964–1,140) constitute the second segment, which exhibits a markedly steeper increase in structural dissimilarity (‘slope2’). Both slopes were statistically significant (p < 0.05), and the ratio of the mean increases between the second and first segments was 0.00099/ 0.00017 = 5.82, indicating that the increase in structural divergence in the second segment was nearly six times greater (see Table 3).

The distribution of individual risk factors across the two segments—based on their presence or absence within the 1,140 evaluated triads—is presented in Table 4. Chi-square testing was used to assess the statistical significance of overrepresentation.

**Table 4:** Frequency distribution of individual risk factors across Segments 1 and 2 based on their presence or absence within all 1,140 triads. Chi-square testing was applied to assess the statistical significance of each variable’s overrepresentation across segments.

Occurrence:	Absence	Presence	Absence	Presence		
Risk Factor/Segment:	1	1	2	2	chi-square	p-value
Age	792	171	177	0	36.98	<0.0001
BMI	797	166	172	5	24.36	<0.0001
Hb	792	171	177	0	36.98	<0.0001
WBC	819	144	150	27	0.01	0.9203
RBC	792	171	177	0	36.98	<0.0001
PLT	797	166	172	5	24.36	<0.0001
UREA	827	136	142	35	3.75	0.0528
CREA	821	142	148	29	0.31	0.5777
GFR	823	140	146	31	1.04	0.3078
GLU	837	126	132	45	17.86	<0.0001
Na+	792	171	177	0	36.98	<0.0001
K+	792	171	177	0	36.98	<0.0001
Cl-	792	171	177	0	36.98	<0.0001
CK-MB mass	838	125	131	46	19.84	<0.0001
hsTnT	846	117	123	54	39.53	<0.0001
D-Dimer	851	112	118	59	55.24	<0.0001
aD-Dimer	836	127	133	44	15.97	0.0001
INR	831	132	138	39	8.13	0.0044
aPTT	843	120	126	51	31.36	<0.0001
CRP	853	110	116	61	62.25	<0.0001

Table 4 presents the distribution of individual risk factors between the two structural segments identified in the taxonomic analysis. Sixteen out of the 20 analyzed variables showed statistically significant differences (p < 0.05) in their occurrence between segments. The most prominent overrepresentation in the high-variability segment was observed for C-reactive protein (CRP), which was present in 61 out of 177 triads in segment 2 (χ² = 62.25, p < 0.0001). Other factors strongly associated with the high-variability segment included D-dimer, adjusted D-dimer, hsTnT, age, hemoglobin, and electrolytes (Na ⁺ , K ⁺ , Cl⁻).

In contrast, white blood cell count (WBC) together with three renal function markers—urea (UREA), creatinine (CREA), and glomerular filtration rate (GFR)—showed no statistically significant difference in distribution between segments (p > 0.05).

Overall, the examined variables exhibited three distinct statistical patterns (Table 4):

- Lack of significant differentiation – parameters whose frequency differences between segments were statistically indistinguishable from random variation in the studied population (WBC, UREA, CREA, GFR).
- Significant predominance in the low-variability segment (segment 1) – factors such as age, BMI, Hb, RBC, PLT, and the electrolyte panel (Na ⁺ , K ⁺ , Cl⁻), whose overrepresentation may reflect a baseline structural configuration observed in the majority of patient triads.
- Significant overrepresentation in the high-variability segment (segment 2) – factors including GLU, CK-MB mass, hsTnT, D-dimer, aD-dimer, INR, aPTT, and CRP, whose clustering within this segment reflects statistically significant deviations from the baseline structure, consistent with a state of increased structural divergence between triads.

## Discussion

In this study, we first applied conventional outcome-based models, including Cox proportional hazard and logistic regression, to identify prognostic factors associated with mortality in acute pulmonary embolism. These analyses confirmed that age was the strongest statistical predictor of death, while several additional biomarkers reached nominal statistical significance. However, this pattern raised an important interpretative problem: age is a non-modifiable background characteristic that reflects accumulated vulnerability rather than acute pathophysiological activity, and the large number of statistically significant predictors suggested substantial redundancy among the modeled variables. In this context, regression alone provided limited insight into which biomarkers were actively involved in the disease process itself, as opposed to merely correlating with outcome.

To address this limitation, we introduced a complementary outcome-agnostic taxonomic framework based on Marczewski–Steinhaus distances. Its purpose was not to infer causality, but to examine how individual variables contribute to the geometric structure of inter-patient variability independently of clinical endpoints. Within this framework, C-reactive protein (CRP) emerged as the most active biomarker in shaping multidimensional patient dispersion, in contrast to age, which primarily promoted structural homogeneity rather than disease-driven divergence. Thus, the taxonomic approach serves as a hypothesis-generating filter, highlighting biomarkers that may carry pathophysiological relevance beyond what is captured by conventional regression models. What else did we learn by applying this original approach? We explain this below.

While this interpretation is quantitatively justified and should be regarded as hypothesis-generating rather than confirmatory, the triadic taxonomic structure of 20 patient characteristics (Fig 2) may be divided into two segments based on a statistically defined change-point in structural variability and corresponding frequency counts (Fig 3, Table 4). Intuitively, the first segment reflects a more homogeneous configuration consistent with a clinically stable or health-related state, whereas the second segment captures increased structural heterogeneity that may be associated with disease-related processes. Within this two-segment framework, three distinct profiles of risk factor behavior can be identified:

- Factors without practical relevance for the clinical assessment of disease, whose signaling of health or illness is neutral and random (p > 0.05). In this study, these include: (WBC, as well as the renal markers UREA, CREA, and GFR).
- Factors insensitive to pathological processes, reflecting stable and enduring patient characteristics more typical of a healthy state rather than a dynamic response to disease (p < 0.05). Among those analyzed, these include: age, BMI, Hb, RBC, PLT, and the electrolytes Na⁺ , K⁺ , and Cl⁻ .
- Factors showing potential susceptibility to the adverse influence of disease and sensitivity to pathological processes, manifested by significant overrepresentation in the diseased structural segment and increased taxonomic dispersion (p < 0.05). These life-threatening signals, in descending order of strength, include: CRP, D-dimer, hsTnT, aPTT, CK-MB mass, GLU, aD-Dimer, and INR.

Moreover, given the nearly sixfold ratio of slope coefficients between segment 2 and segment 1 (see Table 3), one may infer high biochemical activity of the “overrepresented” biomarkers and, consequently, metabolic stimulation of the organism in the inflammatory state compared to the homeostatic state.

Taking regression relationships into account, it appears reasonable to state that age is the strongest predictor of mortality in patients with APE not simply because this follows from calculated statistics (Table 2), but because it is a non-modifiable background determinant of risk rather than a marker of acute pathological processes. This conclusion arises from the geometric structure of this variable in statistical space with other patient characteristics, making patients clinically more similar to one another. Furthermore, by accumulating the burden of years—decline in physiological reserves, comorbidities, and reduced capacity to compensate for acute disturbances—age directly translates into higher mortality regardless of other clinical parameters (e.g., reduced cardiovascular performance, diminished respiratory reserve, slower immune response, impaired metabolic regulation). Thus, while age is the best regression-derived predictor of death (Table 2), it is not a direct signaler of life-threatening risk induced by APE, but rather an indirect one.

In view of the above statistical and clinical considerations, one-dimensional analysis of individual risk factors—e.g., via the coefficient of variation (see Table 1)—appears misleading. Only a combined regression and non-regression approach enables the identification of the most reliable mortality risk factor in APE, which, in light of the presented methodology, is CRP (χ² = 62.25). Next in rank are D-dimer, hsTnT, aPTT, CK-MB mass, GLU, aD-dimer, and INR (with χ² statistics of 55.24, 39.53, 31.36, 19.84, 17.86, 15.97, and 8.13, respectively; see Table 4). Thus, of the initial 15 and 14 biomarkers identified as statistically significant (p < 0.05) in univariate Cox and logistic regression, only GLU, CK-MB mass, hsTnT, D-dimer, INR, and—most strongly—CRP meet the criteria of a risk factor in both analytical approaches.

Notably, elevated CRP levels have previously been associated with higher mortality and adverse outcomes in APE, with thresholds above 48 mg/L [20] and 124 mg/L [21] linked to increased risk of death and hemodynamic instability. CRP has also been consistently associated with elevated 30-day mortality [22,23]. Our findings extend this body of evidence by showing that CRP has the strongest structural contribution to inter-patient variability in multidimensional clinical space, independent of classical regression-based associations. In the case of CRP, its distinct structural behavior may have implications for early risk stratification, patient monitoring, and therapeutic decision-making.

Beyond APE-specific reports, the prominent structural behavior of CRP observed in our taxonomic analysis is consistent with a broader body of evidence linking systemic inflammation to adverse outcomes across cardiovascular and thrombo-inflammatory conditions. In the setting of atrial fibrillation, inflammatory markers have been shown to predict recurrence following ablative procedures; for example, in a recent study comparing inflammatory markers for the prediction of atrial fibrillation recurrence following cryoablation, CRP-related indices were significantly associated with recurrent arrhythmia, underscoring the role of persistent inflammatory activity in arrhythmogenic substrate and disease progression [24]. Similarly, composite indices of systemic inflammation have been associated with non-cardiac complications and poor prognosis in acute coronary syndromes: a study on the systemic immune-inflammatory index demonstrated its predictive value for contrast-induced nephropathy in patients with non-ST-segment elevation myocardial infarction [25], indicating that inflammatory burden may translate into organ vulnerability beyond the primary cardiac event. Moreover, in acute infectious and thrombo-inflammatory contexts such as COVID-19 pneumonia, the C-reactive protein–to–albumin ratio (CAR) has been reported as a strong predictor of in-hospital mortality, reinforcing the prognostic relevance of CRP-based signatures in systemic disease [26].

Taken together, these studies place CRP within a coherent biological framework in which inflammation interacts with thrombosis, endothelial dysfunction, and tissue injury across diverse clinical contexts. From this perspective, our taxonomic findings on CRP do not merely replicate known outcome-based associations but provide an additional structural layer: they indicate that inflammatory activity linked to CRP organizes multidimensional clinical variability in ways that classical regression models do not fully capture. This supports the interpretation that CRP is not only a correlate of clinical severity, but a key informational driver of disease heterogeneity in APE.

To our knowledge, this is the first study to apply a complementary taxonomic, outcome-agnostic approach to risk factor evaluation in APE. Our findings underscore the potential of information-centric metrics to enhance classical models, minimize redundancy, and prioritize variables exhibiting unique structural properties. Importantly, this methodology does not imply causality; rather, it provides a complementary perspective on the informational value of presumed risk factors. Nevertheless, future research should further investigate the biological role of CRP in APE pathogenesis and explore the applicability of this framework in other clinical contexts.

## Conclusions

This study demonstrates that conventional regression analyses may overlook the multidimensional structure of prognostic variables in acute pulmonary embolism (APE). Using a taxonomic, outcome-agnostic approach based on variable triads, we found that C-reactive protein (CRP) emerged as the strongest structural signal among all analyzed factors, displaying a pattern of variability not captured by standard statistical techniques.

Our findings suggest that CRP may carry clinically meaningful information, potentially reflecting underlying inflammatory mechanisms involved in APE progression. Its consistent overrepresentation in high-variability combinations supports the need to reconsider its role—not merely as a passive marker of inflammation, but as a possible contributor to adverse outcomes.

The taxonomic framework introduced here offers a novel and complementary tool for identifying high-value prognostic indicators. By focusing on structural inter-variable relationships rather than direct outcome associations, this method has the potential to enrich clinical insight, reduce redundancy, improve model interpretability, and uncover latent prognostic signals that might otherwise remain hidden in traditional analyses.

The results presented in this study warrant further exploration in future research, including validation in independent datasets and prospective study designs, to expand their clinical applicability.

### Study limitations

This study has several limitations. First, its retrospective design relied on data collected during routine clinical care, which—although complete—was not originally intended for the specific analytical purposes of this study. Second, all participants were drawn from a single tertiary care center, potentially limiting the generalizability of the findings to other populations or clinical settings. Third, the taxonomic method employed is unsupervised and outcome-agnostic; therefore, the segment analysis does not support direct causal inference. Finally, although the geometric approach provides a novel perspective on variable interactions, it requires external validation in independent datasets and prospective cohorts to confirm its reproducibility and clinical relevance.

## Supporting information

S1 File Transparency. (ZIP)
