# Correction: Does C-reactive protein exhibit high prognostic information value in acute pulmonary embolism? A novel structural pathway for disease progression beyond classical statistical associations

**DOI:** 10.1371/journal.pone.0345304

**Published:** 2026-03-17

**Authors:** The PLOS One Staff

In the Materials and Methods section, there is an error in the equation. The division slash (“/”) between |A − B| and max(A, B) is omitted. Please view the complete, correct equation here:


$$\begin{array}{c} \text{D}=|\text{A}-\text{B}|/\text{max}(\text{A},\text{ B}) \end{array}$$

The publisher apologizes for the error.
